# O-Antigen-Dependent Colicin Insensitivity of Uropathogenic Escherichia coli

**DOI:** 10.1128/JB.00545-18

**Published:** 2019-01-28

**Authors:** Connor Sharp, Christine Boinett, Amy Cain, Nicholas G. Housden, Sandip Kumar, Keith Turner, Julian Parkhill, Colin Kleanthous

**Affiliations:** a Department of Biochemistry, University of Oxford, Oxford, United Kingdom; b Wellcome Sanger Institute, Hinxton, United Kingdom; c Macquarie University, Sydney, Australia; d Quadram Institute Bioscience, Norwich, United Kingdom; Princeton University

**Keywords:** bacteriocins, cell envelope, enteric bacteria, outer membrane

## Abstract

The outer membrane of Gram-negative bacteria presents a significant barrier for molecules entering the cell. Nevertheless, colicins, which are antimicrobial proteins secreted by Escherichia coli, can target other E. coli cells by binding to cell surface receptor proteins and activating their import, resulting in cell death. Previous studies have documented high rates of nonspecific resistance (insensitivity) of various E. coli strains toward colicins that is independent of colicin-specific immunity and is instead associated with lipopolysaccharide (LPS) in the outer membrane. This observation poses a contradiction: why do E. coli strains have colicin-expressing plasmids, which are energetically costly to retain, if cells around them are likely to be naturally insensitive to the colicin they produce? Here, using a combination of transposon sequencing and phenotypic microarrays, we show that colicin insensitivity of uropathogenic E. coli sequence type 131 (ST131) is dependent on the production of its O-antigen but that minor changes in growth conditions render the organism sensitive toward colicins. The reintroduction of O-antigen into E. coli K-12 demonstrated that it is the density of O-antigen that is the dominant factor governing colicin insensitivity. We also show, by microscopy of fluorescently labelled colicins, that growth conditions affect the degree of occlusion by O-antigen of outer membrane receptors but not the clustered organization of receptors. The result of our study demonstrate that environmental conditions play a critical role in sensitizing E. coli toward colicins and that O-antigen in LPS is central to this role.

**IMPORTANCE** Escherichia coli infections can be a major health burden, especially with the organism becoming increasingly resistant to “last-resort” antibiotics such as carbapenems. Although colicins are potent narrow-spectrum antimicrobials with potential as future antibiotics, high levels of naturally occurring colicin insensitivity have been documented which could limit their efficacy. We identify O-antigen-dependent colicin insensitivity in a clinically relevant uropathogenic E. coli strain and show that this insensitivity can be circumvented by minor changes to growth conditions. The results of our study suggest that colicin insensitivity among E. coli organisms has been greatly overestimated, and as a consequence, colicins could in fact be effective species-specific antimicrobials targeting pathogenic E. coli such as uropathogenic E. coli (UPEC).

## Abstract (precis)

Escherichia coli infections can be a major health burden, especially with the organism becoming increasingly resistant to “last-resort” antibiotics such as carbapenems. Although colicins are potent narrow-spectrum antimicrobials with potential as future antibiotics, high levels of naturally occurring colicin insensitivity have been documented which could limit their efficacy. We identify O-antigen-dependent colicin insensitivity in a clinically relevant uropathogenic E. coli strain and show that this insensitivity can be circumvented by minor changes to growth conditions. The results of our study suggest that colicin insensitivity among E. coli organisms has been greatly overestimated, and as a consequence, colicins could in fact be effective species-specific antimicrobials targeting pathogenic E. coli such as uropathogenic E. coli (UPEC).

## INTRODUCTION

Bacteria exist in complex communities across a wide range of environments, where the limitation of nutrients such as iron, amino acids, and carbon sources drives competition for survival. In response to such coexistence, bacteria have evolved an arsenal of proteinaceous competition systems, such as colicins, which are proteins secreted by Escherichia coli that kill closely related strains (1). Colicins have a number of properties that make them promising future antimicrobials (2, 3). They are extremely potent; they are active at picomolar concentrations and they are highly species specific, which reduces off-target effects that would otherwise cause dysbiosis (4, 5).

Colicins hijack cell envelope proteins in order to translocate into a cell (1). The archetypal colicin (colicin E9) first binds to an outer membrane receptor (BtuB) before using an intrinsically unstructured domain to thread through the porin OmpF and contact TolB in the periplasm. The Tol complex is then thought to activate colicin import across the outer membrane (OM) in a proton motive force-dependent manner (1, 6, 7). Colicin E9 delivers a DNase toxin to the cytoplasm through a poorly understood interaction with FtsH (8).

The OM is a defining feature of Gram-negative bacteria and acts as a selective permeability barrier, limiting the passage of both hydrophilic and hydrophobic molecules (9). For antibiotics, including bacteriocins, the outer membrane presents a formidable barrier (10). The asymmetric outer membrane contains phospholipid within its inner leaflet, while the outer leaflet consists of lipopolysaccharide (LPS), which has roles in permeability, antibiotic resistance, cell adhesion, and virulence (11–13). LPS has three moieties: lipid A, core oligosaccharides, and the O-antigen. The length of the O-antigen varies considerably between strains depending on the number and size of repeating units; in E. coli, the O-antigen length can vary between 17 and 37 nm depending on the serotype (14).

The O-antigen is not essential and is often lacking in laboratory strains of E. coli, such as K-12. During adaptation to laboratory growth, E. coli K-12 acquired the *rfb-50* mutation through an IS*5* insertion in the *wbbL* gene, which encodes a rhamnosyltransferase (15), resulting in the complete loss of O-antigen and presentation of the rough phenotype. Spontaneous mutations that cause a loss of O-antigen confer sensitivity to colicins, and the length of the O-antigen is known to impact colicin sensitivity (16, 17). A survey of E. coli isolates across multiple culture libraries identified high levels of resistance toward colicins when tested, with >70% of isolates resistant to a single colicin and ∼30% of strains resistant to a panel of 18 colicins (18). Despite the high levels of resistance observed under laboratory conditions, colicin plasmids are widespread across E. coli (10% to 50%) (19). Moreover, colicins have been shown to have an antimicrobial affect against multiple E. coli strains, including uropathogenic E. coli (UPEC) (20).

Approximately 150 million people suffer from urinary tract infections (UTI) each year, of which ∼75% are caused by UPEC strains (21). A clone of E. coli (sequence type 131 [ST131]) identified in 2008 as resistant to fluoroquinolones and positive for CTX-M-15 extended-spectrum β-lactamases is now recognized as a globally disseminated strain and a major lineage within extraintestinal E. coli (ExPEC) (22). UPEC ST131 has become a multidrug-resistant bacterium and a major cause of UTI, particularly in the health care setting (23).

The presence of colicin-expressing plasmids across multiple E. coli strains presents a paradox, since high levels (>70%) of colicin insensitivity are typically observed in the lab (18, 24). Yet, some colicins have been found associated with UPEC strains and have even been proposed as virulence factors (25, 26). Here, we distinguish between specific colicin resistance, through mutations of, for example, colicin receptors, and colicin insensitivity, which is a nonspecific phenotype associated with the cell surface. We show that UPEC ST131 is insensitive to colicins in an O-antigen-dependent manner and that insensitivity can be introduced into K-12 strains by restoring the O-antigen. Critically, however, while O-antigen provides high levels of nonspecific insensitivity, it is dependent on growth conditions and, as such, provides an explanation as to why colicin-expressing plasmids are present in smooth E. coli strains but are not seemingly active against strains when assayed under lab conditions.

## RESULTS AND DISCUSSION

### UPEC ST131 is insensitive to multiple colicins.

**FIG 1:**
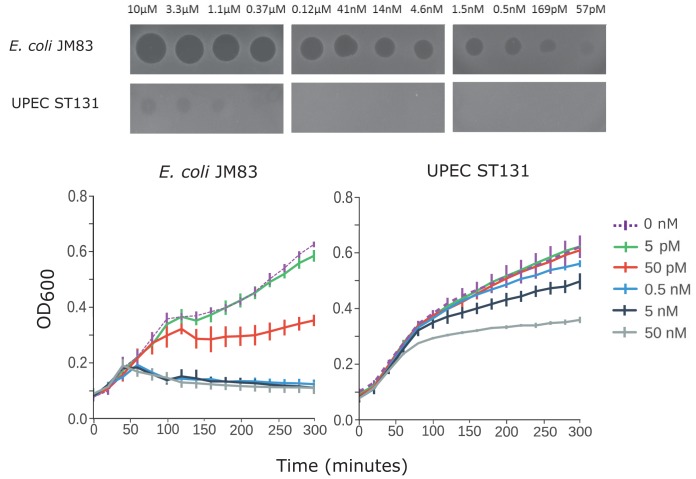
Solid and liquid medium killing assays of UPEC ST131 and colicin-sensitive E. coli JM83 exposed to purified ColE9. (Top) Solid medium killing assays on LB agar using serial dilutions of ColE9. UPEC ST131 shows slight sensitivity to the highest concentrations of ColE9, whereas E. coli JM83 is sensitive at picomolar concentrations. (Bottom) Liquid medium killing assay using LB. UPEC ST131 had a 1,000-fold higher MIC than JM83.

UPEC ST131 exposed to colicin E9 showed a >6,000-fold difference in MIC compared to that of the colicin-sensitive E. coli K-12 strain JM83 when tested on solid medium and a 1,000-fold higher MIC in liquid culture (LB medium) (Fig. 1). Resistance to colicins is typically associated with a cognate immunity protein or mutations in receptor/translocator proteins (1, 27). To test whether these mechanisms were responsible for the observed resistance, UPEC ST131 was exposed to different colicins. The chosen colicins target different receptors/translocation proteins (ColIa, Cir; ColD, FepA; ColE9, BtuB/OmpF), encompassing both the Tol (group A colicins) and the Ton (group B colicins) complexes, and kill the target cell via different mechanisms, including via the depolarization of the cytoplasmic membrane and nucleolytic activity in the cytoplasm (ColIa, pore forming; ColD, tRNase; ColE9, DNase). UPEC ST131 had a 10,000-fold higher MIC of ColIa compared to that of E. coli JM83 and was unaffected by colicin D at the highest concentration tested (10 µM) (see Fig. S1 in the supplemental material). A genome analysis of UPEC ST131 did not identify significant similarity to colicin immunity protein genes for any of the colicins tested, suggesting that mutations in receptors or the presence of immunity proteins is not responsible for the resistance and that UPEC ST131 has a nonspecific mechanism which causes insensitivity to colicin.

To determine if insensitivity toward colicins is a widely observed phenomenon across UPEC strains, 10 UPEC strains were exposed to ColE9 to determine the MICs. Six of the ten strains were not affected by ColE9 at concentrations up to 10 µM (see Tables S1 and S2). The four susceptible isolates had MICs for ColE9 between 30-fold and 2,000-fold higher than the sensitive E. coli MG1655 strain.

### LPS and O-antigen biosynthesis genes are associated with colicin insensitivity.

To elucidate the genetic basis for colicin insensitivity in UPEC ST131, a transposon-directed insertion sequencing (TraDIS) approach was used. TraDIS is a powerful technique to assay the fitness contribution of genes across an entire genome (28). A TraDIS transposon library for UPEC ST131, NCTC 13441, was established using the method described previously (29, 30). The library had a high insertion density, with 450,000 unique insertion sites across the 5.4-Mbp genome, giving an average density of 1 insertion per 12 bp, allowing for an accurate and sensitive evaluation of the role of each gene in colicin insensitivity (barring those which appear essential under these conditions). To test for a role in colicin insensitivity, two samples of the transposon library were prepared: one sample was exposed to a sub-MIC of ColE9 (0.5 nM) and the other was treated with buffer. Genes which provide insensitivity will have a reduced number of transposon insertions compared to that of a control sample, as insertions would disrupt that gene and stop the production of a protective protein. One hundred fourteen genes had a significant decrease in insertions (genes normally involved in insensitivity) compared to that in the non-colicin-treated control, and 34 genes had an increase in insertions (genes normally involved in rendering bacteria susceptible to the colicin) (see Data Set S1). Thirty-three of the genes with decreased insertions (28%) were mapped to five operons responsible for LPS biosynthesis, O-antigen biosynthesis, enterobacterial common antigen (ECA) biosynthesis, membrane lipid asymmetry, and lipid A modification.

The presence of the O-antigen has been shown to provide insensitivity to colicins in some E. coli backgrounds but not for UPEC ST131 (16). Nine of the genes with a role in nonspecific colicin insensitivity are annotated with functions associated with O-antigen production and have been shown to decrease sensitivity toward SDS and serum (31). We also identified seven core LPS genes with roles in colicin insensitivity, in keeping with the O-antigen being attached to core LPS. O-antigen is ligated to core LPS via the O-antigen ligase RfaL, which we also found to be associated with colicin insensitivity. Additionally, *rfaH*, the LPS biosynthesis regulator, was also identified as providing an increase in fitness in the presence of colicin. Four genes of the *arn* operon, involved in the modification of lipid A, were also found to be required for colicin insensitivity. The *arn* operon is involved in resistance to cationic antimicrobial peptides (CAMPs) by increasing the net positive charge of lipid A in the OM through the addition of a 4-amino-4-deoxy-l-arabinose (l-Ara4N) moiety (32). The PhoPQ two-component system is required for activation of the *arn* operon, and both of these components were highlighted as being involved in colicin insensitivity. Increases in l-Ara4N have been shown to increase resistance to polymyxins in Salmonella enterica serovar Typhimurium and Klebsiella pneumoniae (33). The *mla* operon encodes an ATP transporter that is responsible for maintaining the asymmetry of the OM, and mutation of the *mla* genes has been demonstrated to cause sensitivity to SDS and EDTA (34). In addition to the overrepresentation of genes involved in five OM-related operons, we identified 12 genes which had no obvious role in OM stability from their annotation but were shown to have a role in serum resistance in E. coli ST131 (31, 35). Though seven genes from the ECA biosynthesis operon were highlighted by TraDIS, these genes may have roles in O-antigen biosynthesis, and the role of ECA in colicin insensitivity is still to be determined (36). Genes which increased colicin E9 susceptibility included, as expected, those for its receptor and translocator proteins, *btuB* and *ompF*, respectively. Two genes involved in formation of the core LPS, *rfaQ* and *rfaY*, were also identified as increasing colicin susceptibility. Though these genes were not previously identified as necessary for sensitivity to colicins, a positive role of the core LPS in colicin activity has been identified for colicins N and A (37).

### Destabilizing the LPS decreases nonspecific insensitivity toward colicin.

The TraDIS analysis highlighted a significant role for the O-antigen in colicin insensitivity. To test this, we used EDTA to destabilize the LPS packing and assayed for an increase in colicin sensitivity. EDTA destabilizes LPS packing by chelating divalent cations which stabilize adjacent lipid A molecules (38). The introduction of EDTA into the growth medium for UPEC ST131 reduced the MIC for three bacteriocins by 10,000-fold for ColIa and 100-fold for ColE9 (see Fig. S2). UPEC ST131 also became sensitive toward ColD at 10 µM, whereas it previously showed no activity. Repeating the TraDIS analysis in the presence of EDTA (in the media for both the colicin-treated and control pools) revealed 21 genes associated with colicin insensitivity. These genes were all involved in LPS biosynthesis, O-antigen biosynthesis, ECA biosynthesis, and lipid A modification (Table 1).

**TABLE 1:** Genes highlighted by TraDIS as providing a fitness benefit to UPEC ST131 in the presence of colicins^*a*^

Gene name	Function of product	logFC^*b*^	*q* value	Accession no.
0061	LPS core heptosyltransferase	−12.149	1.06E−235	WP_001236430.1
*rfaG_1*	LPS core glycosyltransferase	−2.088	4.55E−029	WP_000634259.1
*rfaH*	Transcription factor involved in LPS biosynthesis	−4.842	1.10E−056	WP_001192400.1
*rfaJ_2*	LPS core glycosyltransferase	−8.354	0	WP_000376840.1
*rfaJ_1*	LPS core 3-alphagalactosyltransferase	−7.005	0	WP_001188027.1
*rfaL*	O-antigen ligase	−5.297	0	WP_000958045.1
*rfbC*	Rhamnose-specific dTDP-4-dehydrorhamnose-3,5-epimerase	−11.967	2.07E−158	WP_001100793.1
*lacA_1*	WbbJ; catalyzes the transfer of the O-acetyl moiety to the O-antigen	−10.953	1.24E−105	WP_001296213.1
*rfbD*	dTDP-glucose-4,6-dehydratase	−10.919	0	WP_000699407.1
*wbbL*	Glycosyltransferase	−10.651	9.64E−080	WP_000262536.1
*rfaG_2*	LPS core glycosyltransferase	−9.977	9.71E−214	WP_001296214.1
01679	Hypothetical protein	−9.190	1.26E−153	WP_000027654.1
01681	Glycosyltransferase	−6.985	2.70E−036	WP_000517629.1
*rfbB*	Rhamnose-specific dTDP-glucose-4,6-dehydratase	−3.545	0	WP_000699407.1
*rmlA1*	Rhamnose-specific glucose-1-phosphate thymidylyltransferase	−2.980	0	WP_000857525.1
*wecA*	ECA transferase	−7.915	0	WP_001050960.1
04729	Putative common enterobacterial antigen polymerase	−7.360	5.76E−048	WP_000055112.1
*wzxE*	ECA flippase	−4.547	1.14E−064	WP_000050265.1
*arnE*	Lipid A modification (CAMP resistance) flippase subunit	−2.413	1.86E−021	WP_000638016.1
*arnD*	Lipid A modification (CAMP resistance) deformylase	−2.271	1.82E−080	WP_000169713.1
02987	Hypothetical protein	−1.960	9.74E−009	WP_001295929.1

a Results are in the presence of EDTA.

b Differences in insertions between the control and colicin-treated sample are measured as a log fold change (logFC).

### O-antigen-restored E. coli K-12 is colicin insensitive.

**FIG 2:**
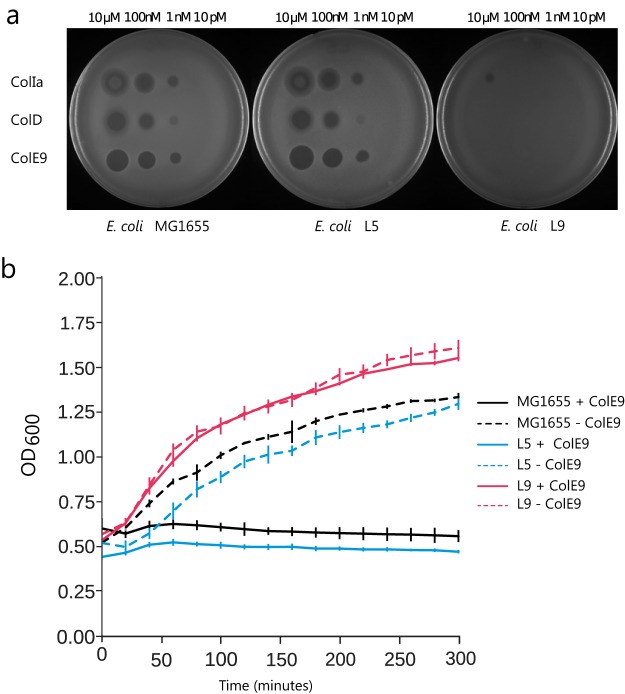
Restoration of the O-antigen in E. coli MG1655 provides insensitivity to a variety of colicins. (a) E. coli strains were exposed to colicins ColIa, ColD, and ColE9, which target different receptor and translocation proteins. (b) Liquid medium killing assay using 50 nM ColE9.

To test directly the effect of the O-antigen on colicin sensitivity, we used strains of E. coli K-12 MG1665 which have a restored O-antigen (39) (Table 2). Henderson and coworkers restored the *wbbL* gene in E. coli K-12 MG1655 to give rise to two strains: L9 with a wild-type (WT) gene order and L5 with incorrect synteny and a significant (∼10-fold) decrease in O-antigen (39). Both MG1655 and the L5 derivative were sensitive to multiple colicins, whereas the L9 strain, with its fully restored O-antigen, was insensitive to 10 µM ColE9 (>100,000-fold increase in MIC) on solid medium and insensitive up to 250 nM ColE9 in liquid medium (>5,000-fold increase in MIC) (Fig. 2). L5 and L9 strains differ only in the densities of O-antigen, demonstrating that the presence of O-antigen is not sufficient to generate colicin insensitivity but that the density of O-antigen is the critical factor for the 100,000-fold increase in MIC. The LPS core has previously been reported as aiding the translocation of colicins N and A (37). These colicins still had a greatly reduced activity toward the L9 strain compared to that toward the L5 strain (see Fig. S3).

**TABLE 2:** Strains used in this study

Strain	Description	Source or reference
E. coli BL21(DE3)	*fhuA2* [*lon*] *ompT gal* (λ) [*dcm*] Δ*hsdS* λ DE3 = λ sBamHIo ΔEcoRI-B *int*::(*lacI*::*PlacUV5*::T7 gene 1) *i21* Δ*nin5*	New England BioLabs
E. coli NEB 5α	*fhuA2* Δ(*argF-lacZ*)*U169 phoA glnV44* ϕ80 Δ(*lacZ*)M15 *gyrA96 recA1 relA1 endA1 thi-1 hsdR17*	New England BioLabs
E. coli MG1655	F^−^ λ^−^ *ilvG*^−^ *rfb-50 rph-1*	
E. coli JM83	F^−^ *ara* Δ(*lac-proAB*) *rpsL* (Str^r^) [ϕ80 *dlac*Δ(*lacZ*)M15] *thi*	
E. coli DFB1655 L5 (L5)	MG1655 with pJP5603/*wbbL* inserted into *rfb* gene cluster	39
E. coli DFB1655 L9 (L9)	MG1655 with pJP5603/*wbbL* inserted into *rfb* gene cluster	39
E. coli UPEC ST131 (NCTC 13441)	CTX-M-15 ESBL	

### Colicin insensitivity in UPEC ST131 is dependent on growth conditions.

The L5 and L9 strains showed that changes in the densities of O-antigen can render bacteria sensitive to colicin. Conversely, destabilization of the LPS packing in the outer membrane by EDTA treatment causes an increase in colicin sensitivity. Given these impacts on colicin sensitivity, we set out to determine if growth conditions influenced colicin sensitivity. Bacteria occupy a wide variety of environmental niches and, as a result, are exposed to different conditions and stresses which could influence the density of O-antigen and therefore colicin sensitivity. Biolog phenotypic microarrays were used to study respiration in the presence and absence of sub-MIC ColE9 across hundreds of different growth conditions (40). Four plates were investigated: two screened various carbon sources, one explored a range of pHs with different additives, and a final plate varied osmolytes and osmolarity (see Data Set S2).

No difference in colicin sensitivity was observed for any of the altered carbon source conditions (191 different conditions). However, colicin sensitivity was found to be dependent on osmolarity and pH. Concentrations of NaCl of >0.68 M caused sensitivity to ColE9 at 41 nM (one-third MIC of ColE9 in Biolog inoculation medium), and sodium lactate (0.62 M) and KCl (>0.4 M) had similar effects. Interestingly, UPEC ST131 was sensitive to sub-MIC ColE9 in the presence of urea. UPEC ST131 was also sensitive to ColE9 at alkaline pH (pH >8). We therefore decided to validate two conditions: Tris and urea. Tris is a commonly used buffer, but it is also known to trigger the release of LPS from the OM (41). Urea was chosen as it is present in the urinary tract as the major constituent of urine (∼150 to 500 mM) (42). UPEC ST131 and the O-antigen-presenting L9 strain were challenged with a sub-MIC of ColE9 (33 nM) in the presence/absence of Tris (100 mM [pH 7.2]) or a physiological concentration of urea (150 mM).

**FIG 3:**
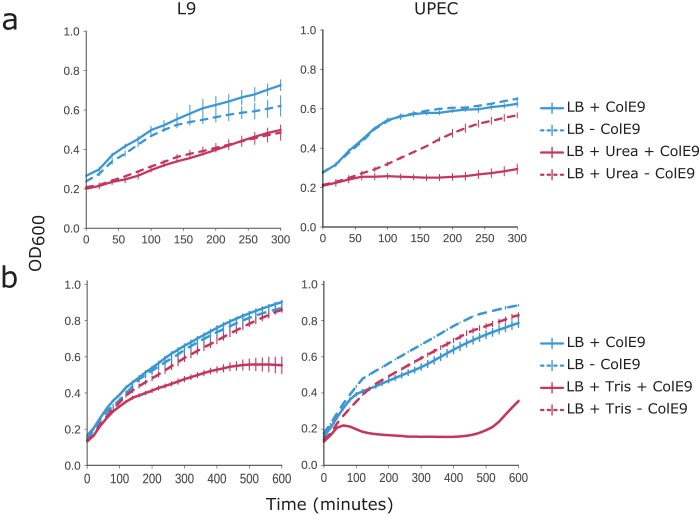
Addition of urea and Tris increases sensitivity toward ColE9 (33 nM) in strains with an O-antigen. (a) Addition of physiological concentrations of urea (150 mM) increased the sensitivity of UPEC ST131 to ColE9 but not that of the E. coli L9 strain. (b) Addition of 100 mM Tris decreased the MICs of ColE9 for both UPEC ST131 and E. coli L9.

The two strains have different O-antigen serotypes (L9, O16; UPEC ST131, O25b) and behave differently under the conditions tested. UPEC ST131 was sensitive to the sub-MIC of ColE9 in the presence of urea and Tris, confirming the Biolog results. In contrast, the L9 strain was only sensitive in the presence of Tris. This may indicate that the L9 strain is producing a denser O-antigen than UPEC ST131 and Tris is a better agent for LPS removal or that UPEC ST131 O-antigen is particularly affected by urea (Fig. 3).

### Dense O-antigen diminishes colicin receptor binding.

Translocation of ColE9 across the outer membrane involves binding to an outer membrane receptor (BtuB) before threading an unstructured domain through OmpF to contact the Tol system in the periplasm (6, 43). O-antigen might provide insensitivity by blocking the binding of ColE9 to its receptor or by preventing the unstructured domain from threading through OmpF. To distinguish between these possibilities, ColE9 binding to BtuB on the surfaces of target bacteria in the presence and absence of O-antigen was investigated by fluorescence microscopy, using Alexa Fluor 647-labeled ColE9 (44).

**FIG 4:**
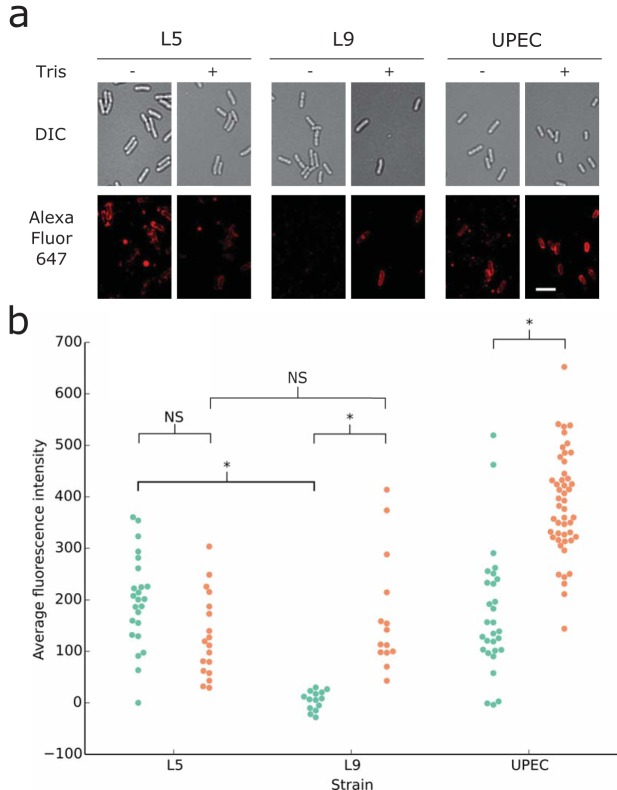
Fluorescently labeled ColE9 shows difference in receptor binding dependent on the density of O-antigen. (a) Colicin E9 labeled with Alexa Fluor 647 (red) was used to visualize the extent of BtuB binding in the OM (45). Scale bar, 5 µm. (b) O-antigen-producing L5 and L9 mutants were compared to UPEC ST131 by exposure to fluorescently labeled ColE9 in LB medium (green) or LB medium supplemented with 100 mM Tris (orange). Each point represents the average fluorescence intensity over a single cell. Statistically significant differences in labeling were observed between the isogenic L5 and L9 strains (*t* test). Addition of 100 mM Tris-HCl caused significant increases in cell labeling for both the L9 strain and UPEC ST131.

A statistically significant difference was observed between the L5 and L9 strains: L5 displayed clear colicin binding, whereas no colicin binding was observed for L9 (Student’s *t* test, *P* < 0.01) (Fig. 4). Tris, which decreases the MIC of colicins for both the L9 strain and UPEC ST131, was evaluated to test if a decrease in MIC correlated with an increase in receptor binding. No differences were observed in colicin binding for the L5 strain in the presence or absence of Tris, suggesting that receptor binding is saturated and the presence of low-density O-antigen is not sufficient to block colicin binding. The addition of Tris caused a significant increase in receptor binding for both the L9 and UPEC ST131 strains. No significant difference was observed in the intensities between the L5 strain and the L9 strain with addition of Tris, suggesting that 100 mM Tris is sufficient to remove any protection afforded by the O-antigen. Urea was not used as a condition for microscopy, as it greatly increased background fluorescence.

Previous studies using fluorescently labeled colicins have shown that outer membrane proteins (OMPs) cluster into supramolecular assemblies termed OMP islands in E. coli lacking O-antigen, which might have effects on the accessibility of OMPs (44, 45). We therefore set out to determine if O-antigen affected this clustering behavior. Using total internal-reflection fluorescence (TIRF) microscopy, we observed OMP islands in the MG1655, L5, and UPEC strains (Fig. S3). The L9 strain could not be validated for the presence of OMP islands, as colicin did not bind BtuB under the conditions tested.

### Concluding remarks.

The presence of O-antigen in the bacterial outer membrane has been linked to nonspecific insensitivity toward colicin, phages, and complement (17, 46). Moreover, the length of O-antigen affects colicin insensitivity in *Shigella* (17). The difference in colicin sensitivity between the E. coli L5 and L9 strains in the present study, coupled with the observation that Tris and EDTA render cells more sensitive to colicin, suggests that subtle changes in the density of O-antigen have significant effects on colicin sensitivity. High levels of colicin resistance across E. coli collections have been observed under lab conditions. Bacteria in the environment will be subjected to a range of stresses and insults to the OM and are unlikely to have an O-antigen as dense as that under laboratory conditions, meaning the scale of colicin insensitivity is likely overestimated. This also suggests that such nonspecific colicin insensitivity could change depending on the environment, host infection site, and stage of infection. For example, during a chronic infection, Pseudomonas aeruginosa loses its O-antigen, while Burkholderia dolosa gains the ability to produce O-antigen (47). For UPEC ST131, the sensitivity toward colicin in the presence of urea presents an intriguing possibility. Uropathogenic E. coli is transmitted to a new host by fecal-oral contamination before mechanical transmission to the urinary tract from the anus (48). UPEC bacteria could therefore be resistant to colicin in the gut, where they will be surrounded by other E. coli, but become sensitive to colicin in the urinary tract, a relatively sterile environment where bacteria would face less competition. It therefore becomes possible that colicins could be an effective treatment for UTI while not affecting commensal E. coli.

Colicin-like proteins are found throughout *Gammaproteobacteria* (24). To date, O-antigen dependent insensitivity has only been studied using colicins. As the O-antigen varies between strains, so may its ability to protect against receptor binding. The O-antigen is not the only structure present at the OM, many bacteria produce a capsule, a thick layer of polysaccharide which can protect against antimicrobial proteins and has been associated with virulence (49). The thickness of this capsule varies greatly between species (E. coli, 10 nm; K. pneumoniae, 160 nm) and could be a significant barrier for colicin receptor binding (50).

Many studies, both theoretical and experimental, have indicated roles for colicins in bacterial competition and in maintaining a diverse microbial community (51, 52). However, it is possible that colicins also have another purpose. In a mixed microbial community of colicin-producing and colicin-insensitive (due to O-antigen) bacteria, the basal level of colicin production would act as a selective pressure to ensure bacteria maintain their O-antigen, increasing the fitness of the overall population.

In summary, we solve the problem of why smooth E. coli harbor colicin plasmids yet appear insensitive toward colicins when assayed under laboratory conditions. O-antigen-dependent colicin insensitivity was observed for UPEC ST131, a clinically important pathogen. Using isogenic O-antigen mutants, we found that the density of the O-antigen is important for colicin sensitivity. Additives that cause shedding of LPS decreased the MIC of colicins for insensitive strains, suggesting that environmental factors influence the density of O-antigen and hence sensitivity toward colicin.

## MATERIALS AND METHODS

### Colicin expression and purification.

Colicin E9 Im9(His)_6_, colicin E9 (H551A K469C) Im9(His)_6_ (53), colicin Ia ImIa(His)_6_, and colicin D ImD(His)_6_ were purified from BL21(DE3) cells induced with isopropyl-β-d-thiogalactopyranoside (IPTG). Proteins were first purified by Ni-affinity chromatography using a 5-ml HisTrap HP column (GE Healthcare) and eluted using a 0-to-250 mM imidazole gradient. After dialysis overnight against 25 mM Tris-HCl (pH 7.5) and 150 mM NaCl, the protein was further purified by size exclusion chromatography using a HiLoad 26/60 Superdex 200 column (GE Healthcare) equilibrated in 25 mM Tris-HCl (pH 7.5) and 150 mM NaCl with the addition of 10 mM dithiothreitol (DTT) for the ColE9 cysteine mutant to prevent dimerization.

### Solid medium killing assay.

Soft LB agar (0.7% [wt/vol)] was inoculated (1:25) with a culture of the indicator strain (optical density at 600 nm [OD_600_] of ∼0.4) and overlaid onto LB agar (1.5% [wt/vol]). Serial dilutions of colicins were prepared using 20 mM Tris-HCl (pH 7), and 2 µl was spotted onto the agar. Plates were incubated overnight at 37°C, and the MIC was defined as the lowest concentration at which a zone of clearance in the soft agar was observed.

### Liquid medium killing assay.

Overnight cultures of the strains E. coli MG1655, E. coli L5, E. coli L9, and UPEC ST131 were used to inoculate (1:100) 10 ml fresh medium supplemented as appropriate and cultured at 37°C until cultures reached an OD_600_ of ∼0.4. Cultures were then diluted (1:3, 200 µl) in a flat-bottomed 96-well plate in triplicates containing medium supplemented with the appropriate additive with or without the addition of colicin. Growth was monitored by measuring the OD_600_ using the Clariostar high-performance microplate reader, and MIC was defined as the concentration at which <50% of growth was inhibited).

### TraDIS library generation, testing, and analysis.

The UPEC ST131 (NCTC 13441) TraDIS library was generated using the method previously described (29). The library was cultured overnight at 37°C in MMTE medium (morpholinepropanesulfonic acid [MOPS] minimal medium with KPO_4_, 1 mM EDTA, 100 mM Tris-HCl [pH 8], and 0.2% [wt/vol] glucose) or MMT medium (MOPS minimal medium with KPO_4_, 100 mM Tris-HCl [pH 8], and 0.2% [wt/vol] glucose) with 0.5 nM ColE9 Im9(His)_6_ or without colicin. Overnight cultures were used to inoculate fresh identical media 1:10 and cultured overnight at 37°C. DNA was extracted from the passaged culture and sequenced using the Illumina HiSeq 2500 platform. Insertions were analyzed using the BioTraDIS pipeline, a pearl library with a host of tools for TraDIS analysis (30). Only reads which contained the transposon tag were considered for analysis. Twenty reads per insertion was considered significant, and reads within 10% of the 3′ end of the open reading frame were ignored, as these insertions may not disrupt protein function (54). A log fold change of >2 with a false-discovery rate (*q* value) of <0.00005 was used as a significant threshold.

### Biolog.

Biolog phenotypic microarrays (PM) monitor the respiration of bacteria across different growth conditions by measuring the reduction of a tetrazolium-based dye (40). Four PM plates were selected for testing: PM1 and PM2, which test different carbon sources; PM9, which tests the effects of osmolytes; and PM10, which tests a range of pH values. UPEC ST131 was cultured overnight in MMTE medium at 37°C overnight with shaking and was used to inoculate the inoculation fluid used in Biolog. Colicin E9 Im9(His)_6_ was added to the PM plates, and as a control, an inactive colicin, which lacks the unstructured region at the N terminus and contains a disulfide bond which does not allow for the conformational change needed for translocation, was used to test for UPEC ST131 respiration without the effect of a cytotoxic colicin (55). Inoculated PM plates were observed by using an OmniLog incubator and reader for 48 h, measuring absorbance every 15 min. Biolog absorbance data were analyzed using the DuctApe PM analysis suite (56). DuctApe measures nine parameters and performs K-means clustering to assign each curve an activity index. Differences between the active- and inactive-colicin-treated clusters were calculated.

### Microscopy and image analysis.

For fluorophore labeling, site-directed mutagenesis was used to incorporate a cysteine into ColE9 (H551A K469C) or ColE9 K469C with an N-terminal deletion, Δ^2-62^, which is known to block import into cells (44). Purified protein was labeled with Alexa Fluor 647 (AF647). AF647 with an exposed maleimide group was covalently linked to the sulfhydryl of the engineered cysteine to form a stable conjugate. Excess dye was removed by passing the protein through a Superdex 75 10/300 column (GE Healthcare).

E. coli L5, E. coli L9, and UPEC ST131 were incubated overnight at 37°C with shaking in LB medium. Overnight cultures were used to inoculate (1:100) LB medium with additives and were incubated at 37°C until an OD_600_ of ∼0.4 was reached. From each sample, 1,600 µl was pelleted by centrifugation at 7,000 × *g* for 3 min, and the pellet was resuspended in 200 µl of 1.5 µM ColE9 and incubated for 30 min with rotary inversion at room temperature. Cells were washed three times in 400 µl of LB with the additive of interest. Cells were resuspended in approximately 200 µl of medium, and 10 µl of cells was placed onto a 1% agar pad. Fluorescence was visualized by widefield microscopy using the DeltaVision OMX V3 system. Images were acquired following excitation at 642 nm for 50 ms and processed using Fiji image processing software (57). Student’s *t* tests were used to identify statistically significant differences between treatment and strains.

### Accession number(s).

TraDIS sequencing data are available under the accessions ERS939266 to ERS939273 and ERS441440 to ERS441443. Other data supporting this study can be found within the article and the supplemental material or requested from the corresponding author.

## Supplementary Material

Supplemental file 1

Supplemental file 2

Supplemental file 3

## References

[1] Cascales E, Buchanan SK, Duché D, Kleanthous C, Lloubès R, Postle K, Riley M, Slatin S, Cavard D 2007 Colicin biology. Microbiol Mol Biol Rev 71:158–229. doi:10.1128/MMBR.00036-06. 17347522 PMC1847374

[2] Behrens HM, Six A, Walker D, Kleanthous C 2017 The therapeutic potential of bacteriocins as protein antibiotics. Emerg Top Life Sci 1:65–74. doi:10.1042/ETLS20160016. PMC7243282 33525816

[3] McCaughey LC, Ritchie ND, Douce GR, Evans TJ, Walker D 2016 Efficacy of species-specific protein antibiotics in a murine model of acute *Pseudomonas aeruginosa* lung infection. Sci Rep 6:30201. doi:10.1038/srep30201. 27444885 PMC4957109

[4] Shannon R, Hedges AJ 1967 Kinetics of lethal adsorption of colicin E2 by *Escherichia coli*. J Bacteriol 93:1353–1359. 5340307 10.1128/jb.93.4.1353-1359.1967 PMC276608

[5] Kageyama M, Kobayashi M, Sano Y, Masaki H 1996 Construction and characterization of pyocin-colicin chimeric proteins. J Bacteriol 178:103–110. doi:10.1128/jb.178.1.103-110.1996. 8550402 PMC177626

[6] Housden NG, Hopper JT, Lukoyanova N, Rodriguez-Larrea D, Wojdyla JA, Klein A, Kaminska R, Bayley H, Saibil HR, Robinson CV, Kleanthous C 2013 Intrinsically disordered protein threads through the bacterial outer-membrane porin OmpF. Science 340:1570–1574. doi:10.1126/science.1237864. 23812713 PMC3856478

[7] Kleanthous C 2010 Swimming against the tide: progress and challenges in our understanding of colicin translocation. Nat Rev Microbiol 8:843–848. doi:10.1038/nrmicro2454. 21060316

[8] Walker D, Mosbahi K, Vankemmelbeke M, James R, Kleanthous C 2007 The role of electrostatics in colicin nuclease domain translocation into bacterial cells. J Biol Chem 282:31389–31397. doi:10.1074/jbc.M705883200. 17720814

[9] Nikaido H 2003 Molecular basis of bacterial outer membrane permeability revisited. Microbiol Mol Biol Rev 67:593–656. doi:10.1128/MMBR.67.4.593-656.2003. 14665678 PMC309051

[10] Ghai I, Ghai S 2018 Understanding antibiotic resistance via outer membrane permeability. Infect Drug Resist 11:523–530. doi:10.2147/IDR.S156995. 29695921 PMC5903844

[11] Walker SL, Redman JA, Elimelech M 2004 Role of cell surface lipopolysaccharides in *Escherichia coli* K12 adhesion and transport. Langmuir 20:7736–7746. doi:10.1021/la049511f. 15323526

[12] Ranf S 2016 Immune sensing of lipopolysaccharide in plants and animals: same but different. PLoS Pathog 12:e1005596. doi:10.1371/journal.ppat.1005596. 27281177 PMC4900518

[13] Band VI, Weiss DS 2015 Mechanisms of antimicrobial peptide resistance in Gram-negative bacteria. Antibiotics (Basel) 4:18–41. doi:10.3390/antibiotics4010018. 25927010 PMC4410734

[14] Strauss J, Burnham NA, Camesano TA 2009 Atomic force microscopy study of the role of LPS O-antigen on adhesion of *E. coli*. J Mol Recognit 22:347–355. doi:10.1002/jmr.955. 19402104

[15] Stevenson G, Neal B, Liu D, Hobbs M, Packer NH, Batley M, Redmond JW, Lindquist L, Reeves P 1994 Structure of the O antigen of *Escherichia coli* K-12 and the sequence of its *rfb* gene cluster. J Bacteriol 176:4144–4156. doi:10.1128/jb.176.13.4144-4156.1994. 7517391 PMC205614

[16] van der Ley P, de Graaff P, Tommassen J 1986 Shielding of *Escherichia coli* outer membrane proteins as receptors for bacteriophages and colicins by O-antigenic chains of lipopolysaccharide. J Bacteriol 168:449–451. doi:10.1128/jb.168.1.449-451.1986. 2428812 PMC213476

[17] Tran EN, Papadopoulos M, Morona R 2014 Relationship between O-antigen chain length and resistance to colicin E2 in *Shigella flexneri*. Microbiology 160:589–601. doi:10.1099/mic.0.074955-0. 24425769

[18] Feldgarden M, Riley MA 1998 High levels of colicin resistance in *Escherichia coli*. Evolution 52:1270–1276. doi:10.1111/j.1558-5646.1998.tb02008.x. 28565393

[19] Riley MA, Wertz JE 2002 Bacteriocin diversity: ecological and evolutionary perspectives. Biochimie 84:357–364. doi:10.1016/S0300-9084(02)01421-9. 12423779

[20] Trautner BW, Hull RA, Darouiche RO 2005 Colicins prevent colonization of urinary catheters. J Antimicrob Chemother 56:413–415. doi:10.1093/jac/dki228. 15980093 PMC2077848

[21] Flores-Mireles AL, Walker JN, Caparon M, Hultgren SJ 2015 Urinary tract infections: epidemiology, mechanisms of infection and treatment options. Nat Rev Microbiol 13:269–284. doi:10.1038/nrmicro3432. 25853778 PMC4457377

[22] Nicolas-Chanoine MH, Bertrand X, Madec JY 2014 *Escherichia coli* ST131, an intriguing clonal group. Clin Microbiol Rev 27:543–574. doi:10.1128/CMR.00125-13. 24982321 PMC4135899

[23] Peirano G, van der Bij AK, Gregson DB, Pitout JD 2012 Molecular epidemiology over an 11-year period (2000 to 2010) of extended-spectrum beta-lactamase-producing *Escherichia coli* causing bacteremia in a centralized Canadian region. J Clin Microbiol 50:294–299. doi:10.1128/JCM.06025-11. 22162555 PMC3264152

[24] Sharp C, Bray J, Housden NG, Maiden MCJ, Kleanthous C 2017 Diversity and distribution of nuclease bacteriocins in bacterial genomes revealed using hidden Markov models. PLoS Comput Biol 13:e1005652. doi:10.1371/journal.pcbi.1005652. 28715501 PMC5536347

[25] Rijavec M, Budic M, Mrak P, Müller-Premru M, Podlesek Z, Zgur-Bertok D 2007 Prevalence of ColE1-like plasmids and colicin K production among uropathogenic *Escherichia coli* strains and quantification of inhibitory activity of colicin K. Appl Environ Microbiol 73:1029–1032. doi:10.1128/AEM.01780-06. 17122402 PMC1800769

[26] Smajs D, Micenkova L, Smarda J, Vrba M, Sevcikova A, Valisova Z, Woznicova V 2010 Bacteriocin synthesis in uropathogenic and commensal *Escherichia coli*: colicin E1 is a potential virulence factor. BMC Microbiol 10:288. doi:10.1186/1471-2180-10-288. 21078157 PMC2995468

[27] Calcuttawala F, Hariharan C, Pazhani GP, Ghosh S, Ramamurthy T 2015 Activity spectrum of colicins produced by *Shigella sonnei* and genetic mechanism of colicin resistance in conspecific *S. sonnei* strains and *Escherichia coli*. Antimicrob Agents Chemother 59:152–158. doi:10.1128/AAC.04122-14. 25331695 PMC4291344

[28] van Opijnen T, Camilli A 2013 Transposon insertion sequencing: a new tool for systems-level analysis of microorganisms. Nat Rev Microbiol 11:435–442. doi:10.1038/nrmicro3033. 23712350 PMC3842022

[29] Langridge GC, Phan MD, Turner DJ, Perkins TT, Parts L, Haase J, Charles I, Maskell DJ, Peters SE, Dougan G, Wain J, Parkhill J, Turner AK 2009 Simultaneous assay of every *Salmonella* Typhi gene using one million transposon mutants. Genome Res 19:2308–2316. doi:10.1101/gr.097097.109. 19826075 PMC2792183

[30] Barquist L, Mayho M, Cummins C, Cain AK, Boinett CJ, Page AJ, Langridge GC, Quail MA, Keane JA, Parkhill J 2016 The TraDIS toolkit: sequencing and analysis for dense transposon mutant libraries. Bioinformatics 32:1109–1111. doi:10.1093/bioinformatics/btw022. 26794317 PMC4896371

[31] Phan MD, Peters KM, Sarkar S, Lukowski SW, Allsopp LP, Gomes Moriel D, Achard ME, Totsika M, Marshall VM, Upton M, Beatson SA, Schembri MA 2013 The serum resistome of a globally disseminated multidrug resistant uropathogenic *Escherichia coli* clone. PLoS Genet 9:e1003834. doi:10.1371/journal.pgen.1003834. 24098145 PMC3789825

[32] Raetz CR, Whitfield C 2002 Lipopolysaccharide endotoxins. Annu Rev Biochem 71:635–700. doi:10.1146/annurev.biochem.71.110601.135414. 12045108 PMC2569852

[33] Olaitan AO, Morand S, Rolain JM 2014 Mechanisms of polymyxin resistance: acquired and intrinsic resistance in bacteria. Front Microbiol 5:643. doi:10.3389/fmicb.2014.00643. 25505462 PMC4244539

[34] Malinverni JC, Silhavy TJ 2009 An ABC transport system that maintains lipid asymmetry in the Gram-negative outer membrane. Proc Natl Acad Sci U S A 106:8009–8014. doi:10.1073/pnas.0903229106. 19383799 PMC2683108

[35] Alexander DC, Valvano MA 1994 Role of the *rfe* gene in the biosynthesis of the *Escherichia coli* O7-specific lipopolysaccharide and other O-specific polysaccharides containing *N*-acetylglucosamine. J Bacteriol 176:7079–7084. doi:10.1128/jb.176.22.7079-7084.1994. 7525537 PMC197083

[36] Allen WJ, Phan G, Waksman G 2012 Pilus biogenesis at the outer membrane of Gram-negative bacterial pathogens. Curr Opin Struct Biol 22:500–506. doi:10.1016/j.sbi.2012.02.001. 22402496

[37] Sharma O, Datsenko KA, Ess SC, Zhalnina MV, Wanner BL, Cramer WA 2009 Genome-wide screens: novel mechanisms in colicin import and cytotoxicity. Mol Microbiol 73:571–585. doi:10.1111/j.1365-2958.2009.06788.x. 19650773 PMC3100173

[38] Leive L 1965 Release of lipopolysaccharide by EDTA treatment of *E. coli*. Biochem Biophys Res Commun 21:290–296. doi:10.1016/0006-291X(65)90191-9. 4159978

[39] Browning DF, Wells TJ, Franca FL, Morris FC, Sevastsyanovich YR, Bryant JA, Johnson MD, Lund PA, Cunningham AF, Hobman JL, May RC, Webber MA, Henderson IR 2013 Laboratory adapted *Escherichia coli* K-12 becomes a pathogen of *Caenorhabditis elegans* upon restoration of O antigen biosynthesis. Mol Microbiol 87:939–950. doi:10.1111/mmi.12144. 23350972

[40] Bochner BR, Gadzinski P, Panomitros E 2001 Phenotype microarrays for high-throughput phenotypic testing and assay of gene function. Genome Res 11:1246–1255. doi:10.1101/gr.186501. 11435407 PMC311101

[41] Vaara M 1992 Agents that increase the permeability of the outer membrane. Microbiol Rev 56:395–411. 1406489 10.1128/mr.56.3.395-411.1992 PMC372877

[42] Liu L, Mo H, Wei S, Raftery D 2012 Quantitative analysis of urea in human urine and serum by ^1^H nuclear magnetic resonance. Analyst 137:595–600. doi:10.1039/c2an15780b. 22179722 PMC4758351

[43] Housden NG, Kleanthous C 2012 Colicin translocation across the *Escherichia coli* outer membrane. Biochem Soc Trans 40:1475–1479. doi:10.1042/BST20120255. 23176501

[44] Rassam P, Copeland NA, Birkholz O, Toth C, Chavent M, Duncan AL, Cross SJ, Housden NG, Kaminska R, Seger U, Quinn DM, Garrod TJ, Sansom MS, Piehler J, Baumann CG, Kleanthous C 2015 Supramolecular assemblies underpin turnover of outer membrane proteins in bacteria. Nature 523:333–336. doi:10.1038/nature14461. 26061769 PMC4905513

[45] Kleanthous C, Rassam P, Baumann CG 2015 Protein–protein interactions and the spatiotemporal dynamics of bacterial outer membrane proteins. Curr Opin Struct Biol 35:109–115. doi:10.1016/j.sbi.2015.10.007. 26629934 PMC4684144

[46] Knirel YA, Prokhorov NS, Shashkov AS, Ovchinnikova OG, Zdorovenko EL, Liu B, Kostryukova ES, Larin AK, Golomidova AK, Letarov AV 2015 Variations in O-antigen biosynthesis and O-acetylation associated with altered phage sensitivity in *Escherichia coli* 4s. J Bacteriol 197:905–912. doi:10.1128/JB.02398-14. 25512310 PMC4325112

[47] Maldonado RF, Sá-Correia I, Valvano MA 2016 Lipopolysaccharide modification in Gram-negative bacteria during chronic infection. FEMS Microbiol Rev 40:480–493. doi:10.1093/femsre/fuw007. 27075488 PMC4931227

[48] Russo TA, Stapleton A, Wenderoth S, Hooton TM, Stamm WE 1995 Chromosomal restriction fragment length polymorphism analysis of *Escherichia coli* strains causing recurrent urinary tract infections in young women. J Infect Dis 172:440–445. doi:10.1093/infdis/172.2.440. 7622887

[49] Campos MA, Vargas MA, Regueiro V, Llompart CM, Alberti S, Bengoechea JA 2004 Capsule polysaccharide mediates bacterial resistance to antimicrobial peptides. Infect Immun 72:7107–7114. doi:10.1128/IAI.72.12.7107-7114.2004. 15557634 PMC529140

[50] Amako K, Meno Y, Takade A 1988 Fine structures of the capsules of *Klebsiella pneumoniae* and *Escherichia coli* K1. J Bacteriol 170:4960–4962. doi:10.1128/jb.170.10.4960-4962.1988. 3049556 PMC211547

[51] Kerr B, Riley MA, Feldman MW, Bohannan BJ 2002 Local dispersal promotes biodiversity in a real-life game of rock-paper-scissors. Nature 418:171–174. doi:10.1038/nature00823. 12110887

[52] Riley MA, Gordon DM 1999 The ecological role of bacteriocins in bacterial competition. Trends Microbiol 7:129–133. doi:10.1016/S0966-842X(99)01459-6. 10203843

[53] -Schneider CG, Penfold CN, Moore GR, Kleanthous C, James R 1997 Identification of residues in the putative TolA box which are essential for the toxicity of the endonuclease toxin colicin E9. Microbiology 143:2931–2938. doi:10.1099/00221287-143-9-2931. 9308177

[54] Griffin JE, Gawronski JD, Dejesus MA, Ioerger TR, Akerley BJ, Sassetti CM 2011 High-resolution phenotypic profiling defines genes essential for mycobacterial growth and cholesterol catabolism. PLoS Pathog 7:e1002251. doi:10.1371/journal.ppat.1002251. 21980284 PMC3182942

[55] Housden NG, Loftus SR, Moore GR, James R, Kleanthous C 2005 Cell entry mechanism of enzymatic bacterial colicins: porin recruitment and the thermodynamics of receptor binding. Proc Natl Acad Sci U S A 102:13849–13854. doi:10.1073/pnas.0503567102. 16166265 PMC1236540

[56] Galardini M, Mengoni A, Biondi EG, Semeraro R, Florio A, Bazzicalupo M, Benedetti A, Mocali S 2014 DuctApe: a suite for the analysis and correlation of genomic and OmniLog phenotype microarray data. Genomics 103:1–10. doi:10.1016/j.ygeno.2013.11.005. 24316132

[57] Schindelin J, Arganda-Carreras I, Frise E, Kaynig V, Longair M, Pietzsch T, Preibisch S, Rueden C, Saalfeld S, Schmid B, Tinevez JY, White DJ, Hartenstein V, Eliceiri K, Tomancak P, Cardona A 2012 Fiji: an open-source platform for biological-image analysis. Nat Methods 9:676–682. doi:10.1038/nmeth.2019. 22743772 PMC3855844

