# Supplementary material for: O-Antigen-Dependent Colicin Insensitivity of Uropathogenic Escherichia coli
Source: J Bacteriol. 2019 Jan 28;201(4):e00545-18. doi: 10.1128/JB.00545-18 (PMC6351738; doi:10.1128/JB.00545-18)
Supplement: Supplemental file 3 [file zjb999094992s3.pdf]

Supplementary Figure 1

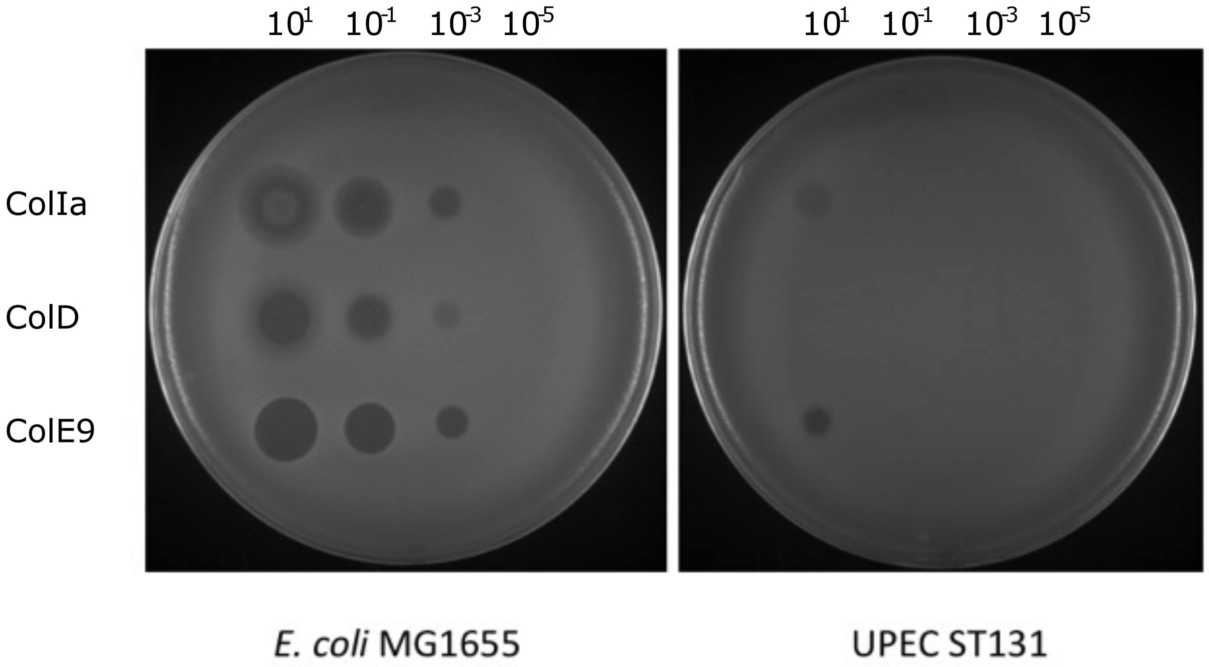

Supplementary Figure 1 UPEC ST131 is insensitive to multiple colicins. Solid media killing assay exposing *E. coli* MG1655 and UPEC ST131 to serial dilutions (begining with 10 µM) of ColIa, ColD and ColE9, three colicins which use different receptors and kill via different mechanisms.

Supplementary Figure 2

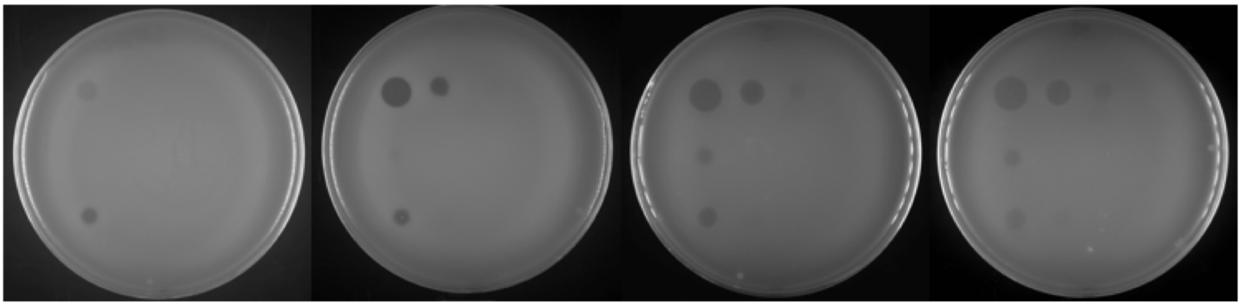

Supplementary Figure 2 UPEC ST131 sensitivity is affected by environmental conditions. Solid media killing assay exposing UPEC ST131 to colicins which target different receptors and kill via different mechanisms. Addition of low concentrations of EDTA (which is known to destabilise LPS) caused ~ 100 fold decrease in MIC for multiple colicin.

Supplementary Figure 3

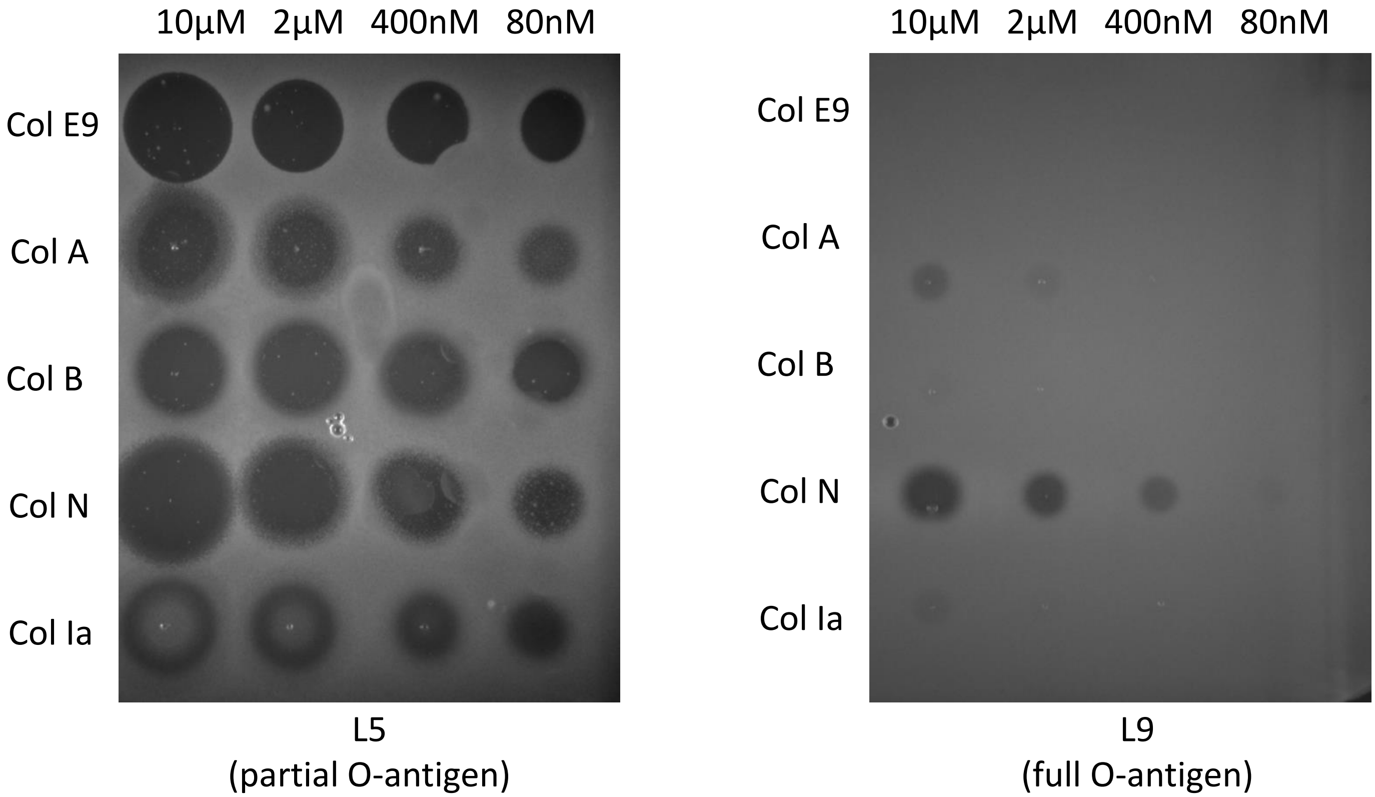

Supplementary Figure 3. Solid media killing assay exposing E. coli L5 and L9 strains (see text) to colicins which target different receptors, have different translocation pathways (Tol- and Ton-) and kill via different mechanisms. Colicins E9, A and N are Tol-dependent while colicins B and Ia are Ton-dependent.

Supplementary Figure 4

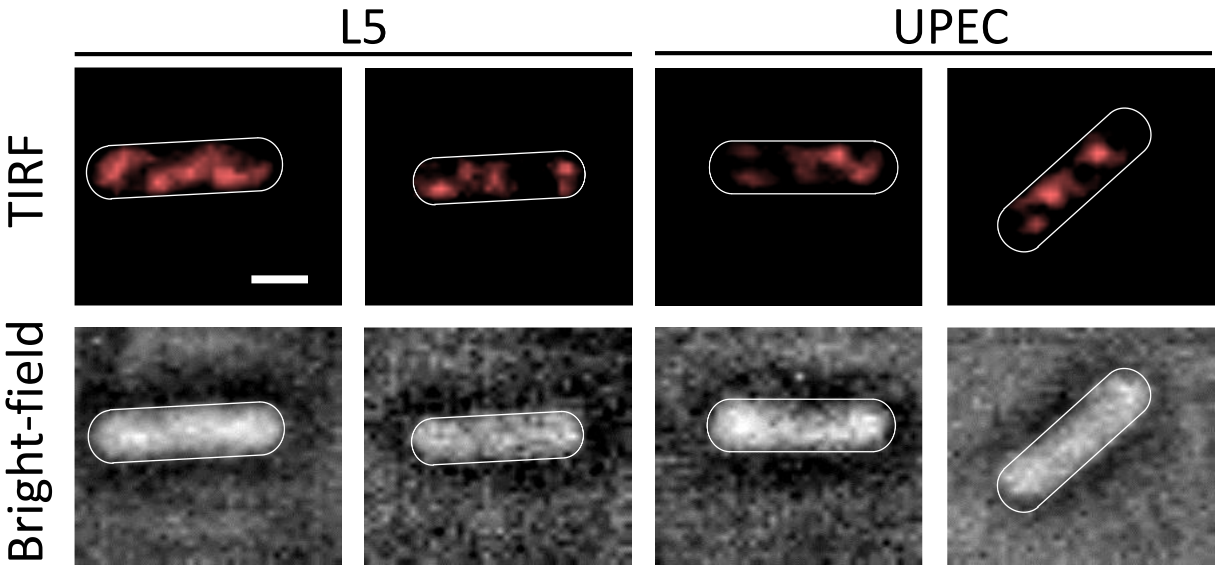

Supplementary Figure 4. TIRF imaging shows non-uniform organization of BtuBs. ColE9-pNGH206 ($\Delta^{2\text{-}61}$ ColE9 K469C) attached to Alexa Fluor-647 was used to label BtuB in exponentially growing L5 and UPEC cells. Images were acquired by TIRF illumination with one 200ms frame. Scale bar indicates 1 µm.

Supplementary Table 1 Plate killing assay of different UPEC strains exposed to different concentrations of Colicin E9 (ColE9). Plus (+) indicates growth of the strain, minus (-) indicates inhibition by the colicin.

ColE9 concentration nM	UPEC isolate										
	2	3	6	8	9	22	34	35	38	50	K-12
10000	-	+	+	+	-	+	-	+	-	+	-
3300	-	+	+	+	-	+	-	+	-	+	-
1100	-	+	+	+	-	+	-	+	-	+	-
370	-	+	+	+	-	+	+	+	+	+	-
120	-	+	+	+	-	+	+	+	+	+	-
41	-	+	+	+	+	+	+	+	+	+	-
14	-	+	+	+	+	+	+	+	+	+	-
4.6	+	+	+	+	+	+	+	+	+	+	-
1.5	+	+	+	+	+	+	+	+	+	+	-
0.5	+	+	+	+	+	+	+	+	+	+	-
0.167	+	+	+	+	+	+	+	+	+	+	+
0.057	+	+	+	+	+	+	+	+	+	+	+

Supplementary Table 2 Details of the different UPEC strains used in this study

Serial No.	Strain designation	IMT-No.	Sequence type	ST Complex	Ancestral Group	Serotype	Host	Year of Isolation	Disease	Sample Accession
**2**	**IMT17424**	17424	10	STC10	A	nt	Pig	2008	UTI	ERS715250
**3**	**IMT8103**	8103	10	STC10	A	O32:H34	Dog	2003	UTI	ERS715251
**6**	**IMT15010**	15010	12	STC12	B2	O18:H6	Dog	2005	UTI	ERS715252
**8**	**764**	6745	14	STC14	B2	O18:H5/11:K5	Human	2003	UTI	ERS715253
**9**	**Ecor64**	7920	14	STC14	B2	O75:NM	Human	unknown	UTI	ERS715254
**22**	**U4252**	13836	48	STC10	A	nt	Human	2001	UTI	ERS715255
**34**	**U5070**	13843	69	STC69	D	nt	Human	2001	UTI	ERS715256
**35**	**IMT14967**	14967	73	STC73	B2	O74:H1	Dog	2005	UTI	ERS715257
**38**	**IMT9258**	9258	73	STC73	B2	O6:K2:H1	human	2004	UTI	ERS715258
**50**	**U3454**	13832	95	STC95	B2	O2:H4	Human	2001	UTI	ERS715259
